# Strategy to combat biofilms: a focus on biofilm dispersal enzymes

**DOI:** 10.1038/s41522-023-00427-y

**Published:** 2023-09-07

**Authors:** Shaochi Wang, Yanteng Zhao, Alexandra P. Breslawec, Tingting Liang, Zhifen Deng, Laura L. Kuperman, Qiuning Yu

**Affiliations:** 1 Otorhinolaryngology Hospital, The First Affiliated Hospital of Zhengzhou University, 450052 Zhengzhou, China; 2 Translational Medicine Center, The First Affiliated Hospital of Zhengzhou University, 450052 Zhengzhou, China; 3 Department of Chemistry and Biochemistry, University of Maryland, College Park, MD 20740 USA; 4 Key Laboratory of Natural Medicine and Immune-Engineering of Henan Province, Henan University Jinming Campus, 475004 Kaifeng, Henan China; 5 Present Address: Mirimus Inc., 760 Parkside Avenue, Brooklyn, NY 11226 USA

**Keywords:** Antimicrobials, Biological techniques

## Abstract

Bacterial biofilms, which consist of three-dimensional extracellular polymeric substance (EPS), not only function as signaling networks, provide nutritional support, and facilitate surface adhesion, but also serve as a protective shield for the residing bacterial inhabitants against external stress, such as antibiotics, antimicrobials, and host immune responses. Biofilm-associated infections account for 65-80% of all human microbial infections that lead to serious mortality and morbidity. Tremendous effort has been spent to address the problem by developing biofilm-dispersing agents to discharge colonized microbial cells to a more vulnerable planktonic state. Here, we discuss the recent progress of enzymatic eradicating strategies against medical biofilms, with a focus on dispersal mechanisms. Particularly, we review three enzyme classes that have been extensively investigated, namely glycoside hydrolases, proteases, and deoxyribonucleases.

## Introduction

### Bacterial Biofilms

Bacterial biofilms consist of surface-attached, and sometimes non-surface attached, colonies embedded within a self-produced extracellular matrix known as the extracellular polymeric substance (EPS). The EPS is composed of extracellular proteins, lipids, nucleic acids (extracellular-DNA and extracellular-RNA), polysaccharides, and secondary metabolites^1–3^ Biofilms are not only capable of reversible surface attachment, but also serve to trap nutrients, as well as shield cells against host immune responses and antimicrobial treatments^4^ Besides these functional roles, the EPS also provides structural support and holds the bacterial cells in close proximity, thereby enabling the exchange of genetic material and facilitating quorum sensing^5,6^ Biofilm-associated infections are common and account for 65-80% of all human microbial infections^7^, such as vaginitis^8^, colitis^9^, conjunctivitis^10^, gingivitis^11^, urethritis^12^, and otitis^13^. Additionally, biofilms formed by adherent bacteria on medical implants and devices can result in serious mortality and morbidity^14^ Furthermore, sessile bacterial colonies covered by established biofilms are more difficult to eradicate than planktonic cells. Biofilms not only function as the physical shield against exogeneous stress, but also lower the metabolic rates of the inhabiting cells to survive harsh environments. As a result, biofilm-associated infections are difficult to eradicate and pose a danger to prevalence of chronic persistentillnesses^15^.

### Biofilm formation

**Fig. 1:**
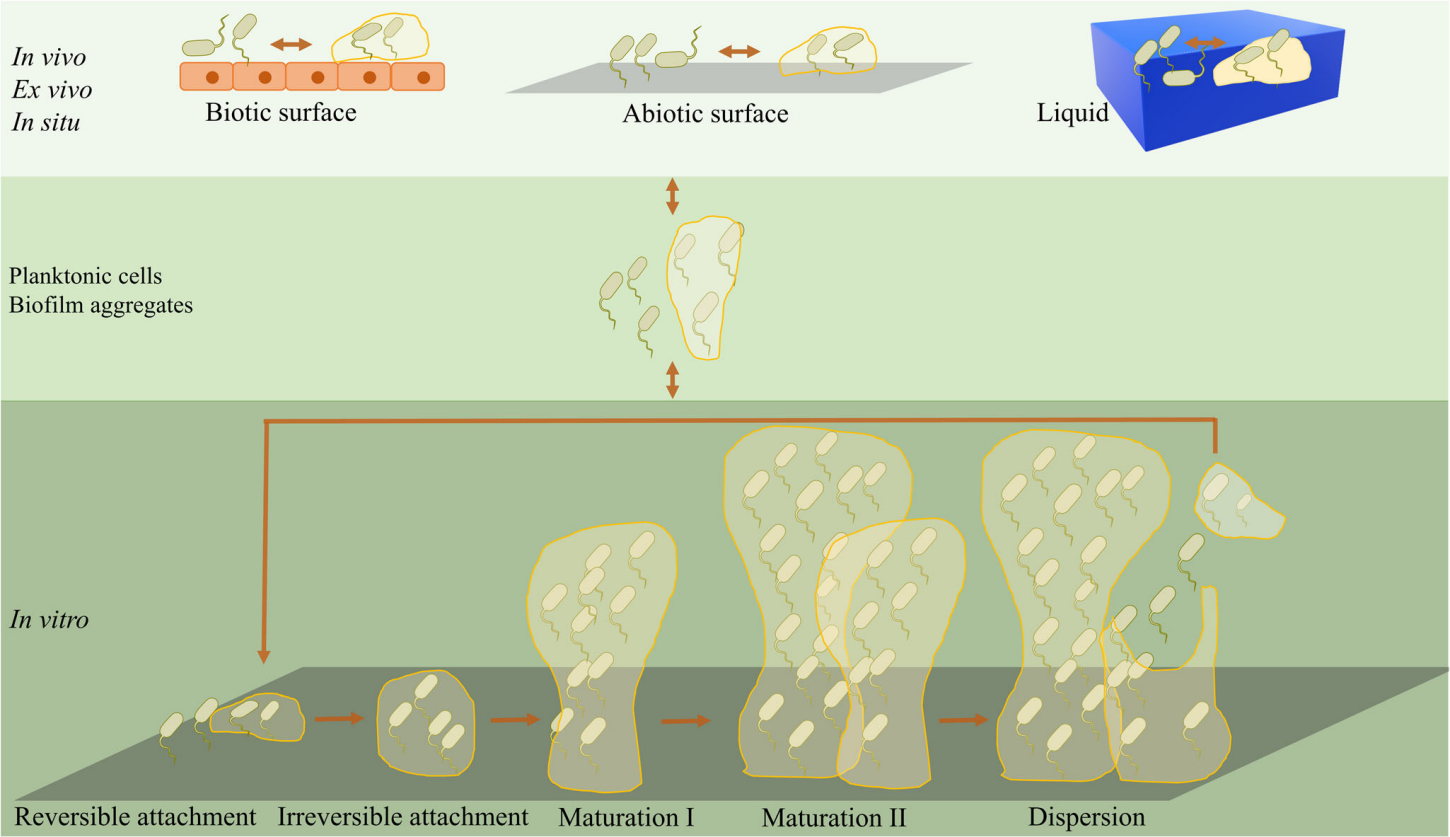
Expanded biofilm formation model. Bacteria can exist as both single cells and biofilm aggregates with regard to environment cues. In vivo, ex vivo, and in situ, bacteria can remain in a planktonic state or reside within non-surface-attached biofilms and these two existing forms are interchangeable depending on the environment. The commonly accepted biofilm formation model, typically the in vitro biofilm developed by *P. aeruginosa*, can be subdivided into five major stages consisting of reversible attachment, irreversible attachment, maturation I, maturation II, and dispersion^16^.

Biofilm formation proves to occur in diverse scenarios and the environment poses a significant influence on biofilm establishment through impacting gene expression and modulating bacteria behaviors. As shown in Fig. 1, an expanded biofilm model was proposed by Sauer et al. and is still growing to reflect all processes involved in the biofilm life cycle^16^. The most commonly accepted model of biofilm formation, typically based on the in vitro biofilm developed by *Pseudomonas aeruginosa*, can be subdivided into five major stages^5^. In the beginning, individual planktonic cells, or preformed aggregates in some cases, migrate and reversibly adhere to a surface. If the surface is suitable for growth, the newly adherent bacterial cells proliferate and initiate biofilm production on the surface. Then, the adherent cells irreversibly attach to the surface, facilitating cell aggregation and EPS production. Later, the biofilm reaches the first stage of maturation (maturation I) and starts to develop mushroom-like structures, which becomes more layered and develop three-dimensional microstructures, including nutrient and water channels. After that, the biofilm reaches a fully mature status (maturation II) with maximal cell density and is now regarded as a three-dimensional community^17^. In the final stage, the mature biofilm releases planktonic cells, with the help of hydrolase enzymes, to migrate and spread to new, unoccupied surfaces^18,19^. However, this model does not entirely represent the complex biofilms formed in the real world including those in industrial, clinical, and natural environments. Indeed, a more inclusive model involving three major events was recently proposed: aggregation, growth, and disaggregation^16^. Besides the in vitro model, biofilms also develop in vivo, in situ, and ex vivo, each of which follows different biofilm developmental pathways in response to diverse environmental factors^16^. In various settings such as on cell surfaces, in fluids, and on transplant devices, surface association is not required and diversely shaped microbial communities are observed. Additionally, in open systems like human gastrointestinal and circulatory systems, there tends to be a consistent influx of new microbial bodies or biofilm aggregates to microbial communities undergoing establishment^16^.

**Fig. 2:**
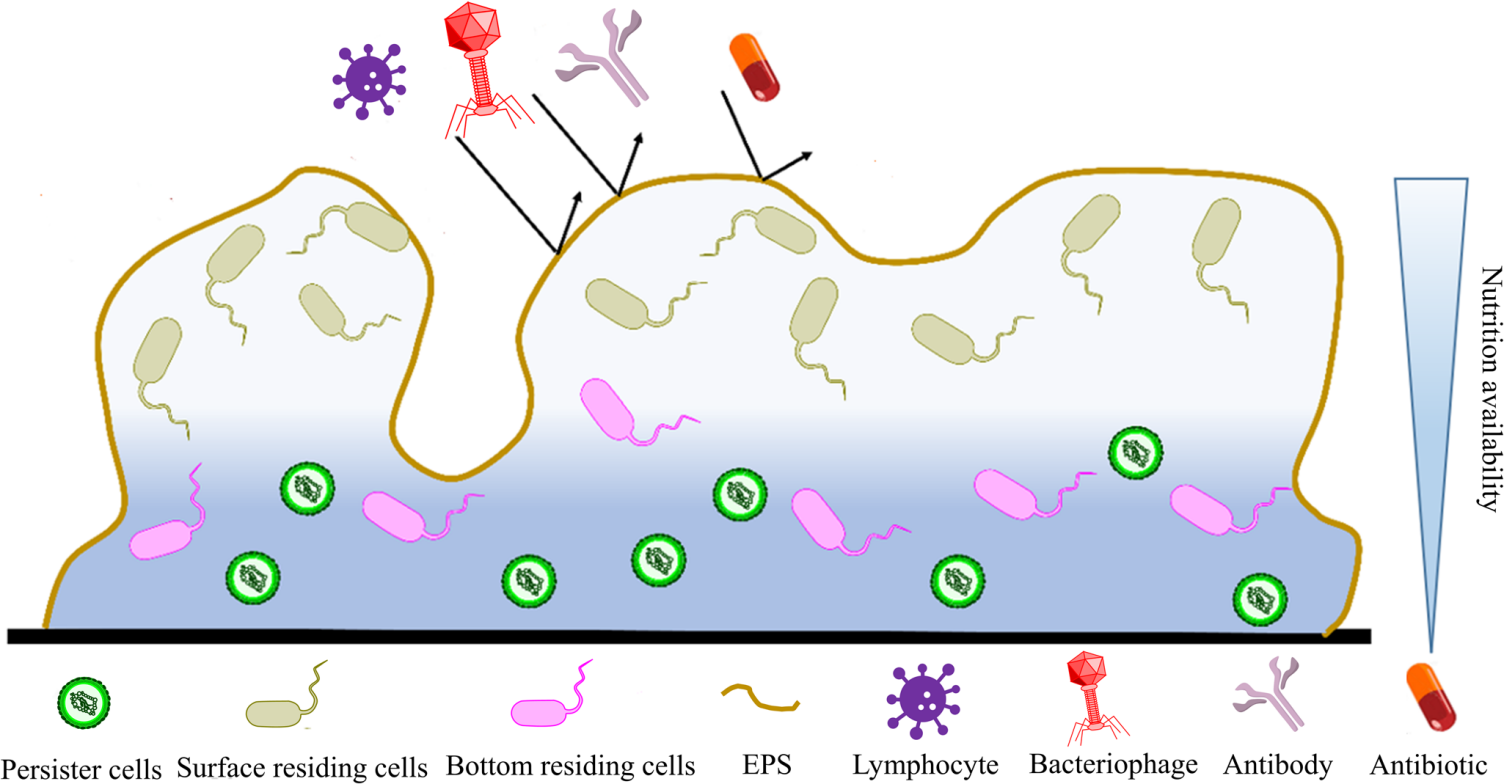
The microbial community enclosed by a biofilm serving as a protective layer against external stress. The biofilm EPS protects the residing bacteria against threats like antibiotics, bacteriophages, and host immune response. While metabolically active surface-residing cells in the nutrition-rich outer portion of the biofilm may be less resistant to environmental pressure, bottom-residing bacteria have greater resistance due to their low metabolic rate. Dormant persister cells can repopulate the bacterial community after antibiotic courses, leading to chronic infections.

Biofilms serve as an effective protective shield for the encased bacterial cells, providing protection from antimicrobial treatments, host immune responses, bacteriophages, and other external stressors, which frequently results in persistent and chronic infections^20^. In fact, studies have shown that bacteria growing in biofilms are often thousands of times more tolerant to antibiotic treatment than their planktonic counterparts^21^. This is in part due to the limited diffusion of nutrients throughout the biofilm EPS resulting in cell heterogeneity (Fig. 2)^22^. Bacterial cells near the biofilm surface are highly metabolically active and more susceptible to antibiotic treatments, while cells in the core of the biofilm exist within a low-oxygen microenvironment, causing these cells to have a decreased metabolic rate, facilitating their resistance to antibiotics^23^. Furthermore, there is a small subpopulation of cells within the biofilm community, known as persister cells, that tend to adopt a dormant state with extreme antimicrobial tolerance^24^. Despite their small numbers, these persister cells contribute significantly to the pathogenesis of biofilm infections^25,26^. Studies indicate that small populations of persister cells are able to survive antimicrobial treatment regardless of the concentration of antibiotic utilized^27^. Once the antibiotic treatment ceases, these remaining persister cells repopulate the microbial community and ultimately lead to a relapsing biofilm infection^28^.

### Biofilm dispersal strategies

There are a number of promising biofilm eradication strategies that have been developed to hinder bacterial biofilm formation or disrupt maturation by dysregulating biofilm growth. These proactive approaches include the use of antimicrobial peptides and lipids^29–31^, medical device surface modifications^32,33^, quaternary ammonium compounds (QACs)^34^, nitric oxide-releasing compounds^35,36^, cell-signaling inhibitors^37,38^, antibiotic-conjugation^39^, and direct surgical removal of biofilm biomass^40^. Biofilm dispersal is an intense area of study that may lead to the development of novel agents that inhibit biofilm formation or promote biofilm cell detachment. Such agents may be useful for the prevention and treatment of biofilms in a variety of industrial and clinical settings^41^.

In clinical settings, enzymes, small molecules, surgical removal, and other strategies have been successfully applied to break down biofilms and release microbes to a more vulnerable planktonic state^42^. Thus, dispersal agents are utilized to improve therapeutic outcomes by increasing access of antimicrobials and host immune cells to the bacteria^43^. Compared with other biofilm dispersal strategies, enzymatic treatments have more advantages. Biofilm-dispersing enzymes are more effective on both growing and pre-existing biofilms, and relatively low concentrations are required to achieve high specificity and efficacy towards the targeted biofilms. Additionally, antibiotic resistance, the issue that many small molecule drugs face, is a less likely occurrence for biofilm-dispersing enzymes, which function extracellularly without the need to be transported across the outer membrane.

### Biofilm dispersal enzymes

Extracellular enzymes can effectively disperse bacterial biofilms by degrading the EPS, specifically by targeting exopolysaccharides, extracellular DNA, and extracellular proteins within in the EPS. By hydrolyzing the microbe biofilm, these enzymes initiate the detachment of sessile bacterial cells and convert them to a planktonic state, which causes increased susceptibility to antibiotics and the host immune system. By laboratory approaches of isolation or over-expression in model organisms, biofilm-dispersing enzymes can be procured at high concentrations and added exogenously to microbial colonies to efficiently break down biofilms. Herein, we review the recent progress of biofilm disruption via three major enzyme classes: glycoside hydrolases^44,45^, deoxyribonucleases^46,47^, and proteases^48,49^.

### Exopolysaccharides within the EPS

**Fig. 3:**
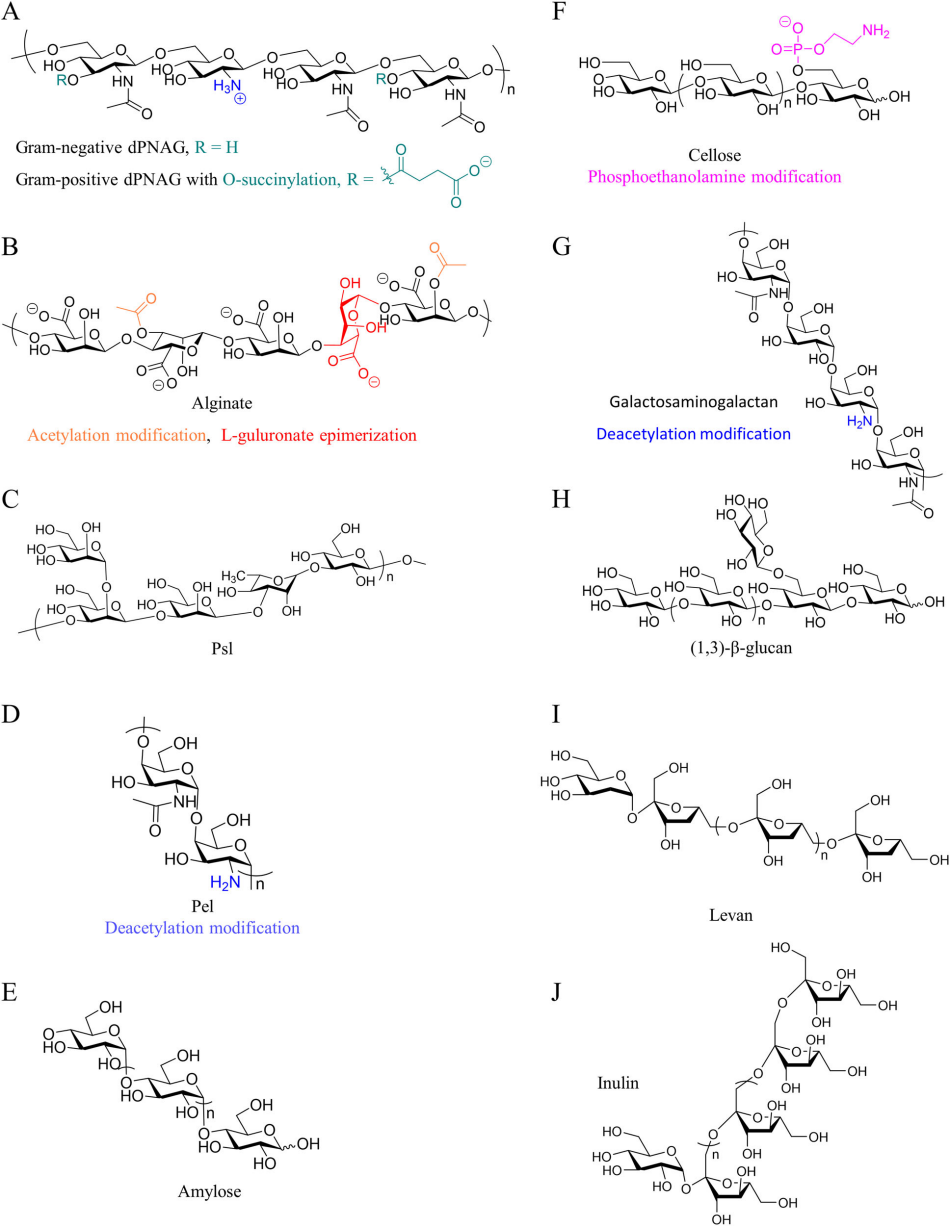
Structures of the most common biofilm exopolysaccharides. dPNAG (**A**), alginate (**B**), Psl (**C**), Pel (**D**), amylose (**E**), cellulose (**F**), galactosaminogalactan (**G**), β-(1,3)-Glucan (**H**), levan (**I**), and inulin (**J**), produced by various bacterial species. The important chemical modifications are colored.

As the major component of the EPS, secreted extracellular polysaccharides are critical for biofilm integrity. Exopolysaccharides widely exist as structural components in microbial biofilms including poly-*N*-acetylglucosamine (dPNAG), alginate, Psl, Pel, amylose-like glucan, cellulose, galactosaminogalactan, β-(1,3)-glucan, levan, and inulin (Fig. 3)^50–54^.

#### dPNAG Exopolysaccharide

Many medically relevant microbial pathogens produce a common exopolysaccharide, partially de-*N*-acetylated poly β-(1,6)-*N*-acetyl-d-glucosamine (dPNAG), as a key component of their biofilm matrix (Fig. 3A)^55^. Both Gram-positive and Gram-negative bacteria have been confirmed to produce dPNAG (sometimes referred to as polysaccharide intercellular adhesin in Gram-positive strains) as a biofilm exopolysaccharide, including *Staphylococcus aureus*^56^, *Escherichia coli*^57^, *Yersinia pestis*^58^, *Actinobacillus pleuropneumoniae*^59^, *Aggregatibacter actinomycetemcomitans*^60^, *Bordetella* species^61^, *Acinetobacter baumannii*^62^, *Burkoholderia* species^63^, *Klebsiella pneumoniae*^64^, *Vibrio parahaemolyticus*^65^, and *Bacillus subtilis*^66^. Individual dPNAG polysaccharides are tens to hundreds of monosaccharide units in length^67^. In Gram-positive bacteria, the *icaABCD* locus is responsible for dPNAG production, whereas Gram-negative bacteria the homologous *pgaABCD* operon regulates its formation^54,68,69^. Chemical modifications of dPNAG, such as *N*- deacetylation and *O*-succinylation, play key roles in the adhesiveness and structural integrity of the biofilm matrices^70^. In Gram-positive bacteria, IcaB is responsible for the *N*-deacetylation of PNAG, and *O*-succinylation of PNAG is catalyzed by IcaC^71^. In Gram-negative bacteria, PgaB C-terminal domain functions as *N*-deacetylase towards PNAG polymers^72^.

#### Alginate exopolysaccharide

Alginate was the first and most thoroughly studied biofilm exopolysaccharide discovered and is produced by *P. aeruginosa*, a pathogenic bacterial species associated with lung infections in cystic fibrosis patients^73^. Alginate is composed of β-d-mannuronic acid and its C-5 epimer, α-l-guluronic acid, connected through (1,4)-glycosidic linkages (Fig. 3B). Most of the enzymes responsible for alginate biosynthesis are encoded by the *alg* operon (*algACD844KEGXLIJF*) in the *P. aeruginosa* genome^74^. The synthesis of the sugar-nucleotide precursors of alginate require the *algACD* operon; *algA* and *algD* are found on the alginate operon while *algC* is located in the genome at PA5322^75^. Chemical modifications are commonly found at the C-2 and C-3 positions of mannuronate residues in alginate polymers. They are frequently acetylated, which is driven by the combined effect of the acetyltransferases AlgI, AlgJ, AlgF and AlgX with varied acetylation rates from 4 to 57%^76,77^. In addition to acetylation, AlgG also catalyzes the epimerization of β-d-mannuronic acid to α-l-guluronic acid^78^. Alginate can facilitate the formation of gel-like structures in the presence of cations, including sodium and calcium, with functional properties strongly correlated to the ManA/GulA ratio and sequence^79^.

#### Psl exopolysaccharide

Psl exopolysaccharide serves as structural scaffold, and plays a key role in surface attachment and eDNA interactions in the biofilm matrix of the opportunistic pathogen, *P. aeruginosa*^80^. The Psl exopolysaccharide contains a penta-saccharide repeating unit consisting of d-mannose, l-rhamnose, and deoxyglucose (Fig. 3C). The biosynthesis of the Psl exopolysaccharide occurs via a Wzx/Wzy-dependent mechanism and is accomplished by 12 proteins encoded by the *pslABCDEFGHIJKL* operon^73^.

#### Pel exopolysaccharide

Pel is one of the most phylogenetically widespread biofilm matrix determinants in both Gram-negative and Gram-positive bacteria (Fig. 3D)^81^. A recent study shows that Pel is a partially de-*N*-acetylated linear polymer of α-1,4-*N*-acetylgalactosamine, comprised predominantly of dimeric repeats of galactosamine and *N*-acetylgalactosamine^82^. Gram-negative bacteria, *P. aeruginosa*, forms Pel-dependent biofilms regulated by a seven gene operon (*pelABCDEFG*), whereas numerous Gram-positive bacterial species use a variant form of this gene cluster (*pelDEA*_*DA*_*FG*) to produce Pel-like polysaccharide^83–85^. In *P. aeruginosa* biofilms, *PelDEFG* mediates sugar polymerization and transport across the cytoplasm, while PelBC is responsible for export^83,86^. PelA exhibits hydrolase and deacetylase activities and regulates the deacetylation of Pel polymers^87^.

#### Amylose-like glucan

Gram-negative bacterial species, such as *Francisella tularensis* and *Pasteurella multocida*, produce biofilm matrices containing amylose-like glucan, an exopolysaccharide made of α-d-glucose units connected through α-(1,4) glycosidic bonds (Fig. 3E)^88,89^. In the production of capsular polysaccharide (CPS) by *P. multocida* biofilms, which consist of amylose-like glucan, capsular polysaccharide production was found to be inversely related to biofilm formation^89^. Little is known about the genes of amylose exopolysaccharides; more work is needed to reveal its biosynthetic mechanism.

#### Cellulose exopolysaccharide

Cellulose, composed of β-(1,4)-d-glucose (Glc) monomer subunits (Fig. 3F), has been identified as a biofilm matrix component of several bacterial species including *Agrobacterium tumefaciens*^90^, *Escherichia coli*^91^, *Pseudomonas flurescens*^92^, and *Gluconacetobacter xylinus*^51^. The cellulose biosynthetic and secretive machineries of various bacteria are extremely diverse, and different bacteria utilize varying bacterial cellulose synthase (*bcs*) operons to produce this exopolysaccharide^93^. Multiple chains of cellulose can begin to form greater aggregates through hydrogen bonding interactions between cellulose polymer strands^94^. Besides this, phosphoethanolamine-modified cellulose generated by *E. coli* is required for extracellular matrix assembly and biofilm architecture^95^. The modification is catalyzed by phosphoethanolamine transferase, BcsG, in the presence of biofilm-promoting cyclic diguanylate monophosphate^95^.

#### Galactosaminogalactan

Galactosaminogalactan (GAG), commonly found in the biofilms of various fungal species, is a heteroglycan composed of galactose and *N*-acetylgalactosamine (GalNAc) linked by α-(1,4) glycosidic bonds (Fig. 3G)^96^. In biofilm-associated infections, GAG serves as an adhesion factor to the host, and mediates virulence by masking other pathogen-associated molecular patterns (PAMPs). The synthesis of GAG is regulated by a cluster of genes *(gtb3, agd3, ega3, sph3*, and *uge3*) encoding five eponymic, carbohydrate-active enzymes^97^. Agd3, categorized as a carbohydrate esterase family CE18 enzyme, deacetylates GAG in a metal-dependent manner^98^. Deacetylation of GAG serves as a key factor for adherence to hyphae and mediates biofilm formation^97^.

#### β-(1,3)-Glucans

β-(1,3)-glucans are glucose polymers mainly linked by β-(1,3)-glycosidic bonds with branched side chains attaching to the backbone through 1,6-linkages (Fig. 3H)^99^. Synthesis of the linear β-(1,3)-glucan polymer is catalyzed by UDP-glucose glucosyltransferase in many microbial species including *Candida albicans*, *Aspergillus fumigatus* and *Cryptococcus neoformans*^100^. β-(1,3)-glucans are the primary components of the *C. albicans* biofilm EPS and are important for *C. albicans* biofilm formation and stress resistance^101^.

#### Fructan exopolysaccharide

Levan and inulin (Fig. 3I-J) are two primary fructans discovered in many microbial biofilms including the genera *Acetobacter, Bacillus, Erwinia, Gluconobacter, Halomonas, Microbacterium, Pseudomonas, Streptococcus*, and *Zymomonas*^102^. Levan is composed of β-(2, 6) glycosidic fructosyl bonds with occasional β-(2, 1) branching^103^, while inulin is primarily comprised of β-(2, 1) fructosyl linkages and some β-(2, 6) linkages at the branching point^104^. Microbial levan is synthesized through transfructosylation by a secreted levansucase (EC: 2.4.1.10) from sucrose substrates in *Bacillus* species^105^.

These exopolysaccharides play important roles in biofilm establishment and persistence through enhancing structural stability, defense against environmental stress, adhesion and aggregation of cells, absorption of exogenous compounds, and providing a carbon source during starvation. Because of their indispensable function in biofilm integrity, glycosidase enzymes that target exopolysaccharides are emerging as an effective means to disperse biofilms^106–108^. The glycol-hydrolases discussed are in the same order as the introduced corresponding exopolysaccharides.

### Glycoside hydrolase enzymes

#### dPNAG glycoside hydrolase—Dispersin B

Dispersin B (DspB) belongs to glycoside hydrolase family 20 (GH20) and was first isolated from *Aggregatibacter actinomycetemcomitans*^109^. DspB is known to hydrolyze the exopolysaccharide dPNAG in biofilm matrices through both *endo-* and *exo*-glycoside hydrolase activity^110–112^. DspB utilizes a substrate-assisted mechanism in dPNAG hydrolysis in which the substrate’s 2-acetamido group facilitates glycoside hydrolysis through formation of a characteristic oxazolinium ion intermediate^113^. Within the catalytic site, the amino acid residue, D183, serves as catalytic acid and D184 stabilizes the oxazolinium ion intermediate^113^. In vitro studies show that DspB can effectively disperse biofilms formed by bacteria like *S. aureus*, *A. actinomycetemcomitans*, *S. epidermidis*, *A. baumannii*, *K. pneumoniae*, *E. coli*, *Burkholderia* spp., *A. pleuropneumoniae*, *Y. pestis*, and *P. fluorescens*. In an in vivo study, DspB was prepared into DispersinB® wound gel by Kane Biotech Inc., which significantly accelerated the healing of both infected and non-infected dermal wounds compared to controls^114^. Compared with wild-type DspB, most DspB mutants present significantly reduced activity on synthetic PNAG probes^106,107^. However, DspB_E248Q_ demonstrates remarkably increased dPNAG breakdown and effective dispersal of *S. aureus* preformed biofilms^115^.

#### dPNAG glycoside hydrolase—PgaB

PgaB is a glycoside hydrolase encoded by the PNAG biosynthetic operon, namely by the gene *pgaB*, and has the capability to degrade PNAG synthetic analogues, as well as disrupt PNAG-dependent biofilms formed by *Bordetella pertussis, Staphylococcus carnosus, S. epidermidis*, and *E. coli*^116,117^. PgaB is a two-domain periplasmic protein that contains an N-terminal deacetylase domain that regulates PNAG deacetylation and a C-terminal PNAG binding domain that modulates PNAG export^118^. Detailed analysis shows that PgaB contains a C-terminal CAZy GH153 family glycosyl hydrolase that catalyzes the endoglycosidic cleavage of dPNAG containing de-*N*-acetylated glucosamine (GlcN) in the −3 binding site^116^. The C-terminal domain of PgaB produced by *Bordetella bronchiseptica* has a central cavity within an elongated surface groove that preferably recognizes the GlcN-GlcNAc-GlcNAc motif (where GlcNAc is *N-*acetylglucosamine), and the catalytic site amino residue, D474, functions as a catalytic acid to digest the dPNAG substrate. After hydrolysis, mass spectrometry reveals the GlcN-GlcNAc-GlcNAc motif at the new reducing end^116^. This research shows that PgaB not only serves as a deacetylase within the PNAG biosynthetic machinery, but also possesses glycoside hydrolase activity and may be used as a therapeutic agent against PNAG-dependent biofilm infections^116^.

#### Alginate glycoside hydrolase

In addition to dPNAG hydrolases, alginate lyase enzymes have been shown to exhibit effective dispersal of mature biofilms^119^. Alginate lyases catalyze the degradation of alginate, and have been isolated from various organisms with different substrate specificities, including algae, marine mollusks, marine and terrestrial bacteria, and some viruses and fungi^120^. Many studies demonstrating the antibiofilm activity of alginases have used crude cell extracts from *Flavobacterium multivorum*, but the synergistic effect with antibiotics remains contradictory^121^. Two distinct alginate lyase enzymes in *F. multivorum* extract have been discovered and characterized: one of which exhibits degradation towards both poly-β-d-mannuronate (polyM) and poly-α-l-guluronate (polyG), while the other only has polyG degradation activity^119^. Only alginate lyase enzymes with polyM/G activity are effective in destroying preformed mature biofilms and have a synergistic effect with antibiotics^119^. A recent study shows that purified marine alginate lyase enzyme (AlyP1400) is able to degrade *P. aeruginosa* biofilms and enhances bactericidal activity of the antibiotic, tobramycin, while also modulating expression of efflux antibiotic resistance-related genes; *bdlA*, *mexF*, *mexY*, and *ndvB*; suggesting an increased susceptibility of *P. aeruginosa* biofilms to this combinatorial treatment^122^.

#### Psl glycoside hydrolase

PslG, a member of glycoside hydrolase family 39 (GH39), is periplasmic glycoside hydrolase encoded by the Psl exopolysaccharide biosynthetic operon^123^. After removal of the N-terminal transmembrane domain, PslG_h_ (which has a soluble catalytically active glycoside hydrolase domain) can hydrolyze Psl in *P. aeruginosa* biofilms^123^. PslG_h_ inhibits clinical and environmental isolates of *P. aeruginosa* biofilm formation over a 24-h period and is also capable of disrupting newly formed biofilms but is less potent to disperse mature biofilms. Further, PslG_h_ can potentiate the antibacterial effect of colistin, an antibiotic used to treat Gram-negative multi-drug resistant infections^124^. PslG_h_ is noncytotoxic and support immune defenses; the enzyme does not impact host cell morphology and enhances neutrophil killing activity^124^.

#### Pel glycoside hydrolase

PelA, a periplasmic glycoside hydrolase encoded in the Pel exopolysaccharide biosynthetic operons, contains at least two catalytic domains—a putative glycoside hydrolase domain and a CE4 deacetylase domain^87^. Based on a bioinformatic analysis. the N-terminal domain of PelA was removed, generating the PelA_47–303_ construct (referred as PelA_h_), was expressed and purified in a study of its glycoside hydrolase activity^124^. Prophylactic treatment with PelA_h_ resulted in a 2.5-log reduction of *P. aeruginosa* bacterial colony-forming units, and application of PelA_h_ to established biofilms resulted in significant biofilm dispersal within 24 h^124^. Furthermore biofilm disruption with PelA_h_ is not sensitive to the maturation state of the biofilm^124^. PelA_h_ also boosted the antibiotic efficacy of colistin and increased neutrophil killing by ~50%^124^.

#### Amylose glycoside hydrolase

Endo-acting α-amylase, of the glycoside hydrolase family 13 (GH13), cleaves α-(1,4)-d-glucosidic linkages at random sites of amylose exopolysaccharide in biofilm matrices leading to biofilm dispersing events^125^. Research shows that α-amylase from *Aspergillus oryzae, Bacillus subtilis*, human saliva, and sweet potato demonstrates a strong inhibiting effect on *S. aureus* biofilm buildup, as well as degrade existing pre-formed *S. aureus* biofilms^126^. However, a less severe inhibiting effect was observed for β-amylase from sweet potato (~50% inhibition versus 77-89% inhibtion from the others) because it is an exo-acting GH14 carbohydrolase which hydrolyzes the α***-***1,4-glucosidic linkages of amylose exopolysaccharide only from the nonreducing end^126^.

#### Cellulose glycoside hydrolase

Cellulase is a glycoside hydrolase produced chiefly by fungi, bacteria, and protozoans that acts specifically by breaking down the β-(1,4) linkages in polysaccharides, such as cellulose, an exopolysaccharide commonly found in the biofilm of several bacteria, including *E. coli*, *Salmonella*, *Citrobacter*, *Enterobacter*, and *Pseudomonas* as well as *Agrobacterium tumefaciens*^127^. Cellulase from various sources such as *Penicillium funiculosum* and *Trichaderma reseei* can inhibit biofilm formation of *P. aeruginosa* in a pH dependent manner, in which exogenously added cellulase is more effective at pH 5 than pH 7^128^. Treatment combining cellulase with ceftazidime, an antibiotic, can more effectively inhibit *P. aeruginosa* biofilm formation and attachment^129^. In vitro testing also shows that Levofloxacin, an antibiotic for severe infection, combined with cellulase can powerfully disperse mature biofilms formed by *bacille CalmetteGuerin*^130^.

#### Galactosaminogalactan glycoside hydrolase

Sph3 is encoded by the *Sph3* gene, which belongs to the five gene cluster regulating GAG biosynthesis. The glycol-hydrolase domain (Sph3_h_) of Sph3 is classified as glycoside hydrolase family 135 (GH135)^131^. Sph3 has the (β/α)_8_ fold structure that many glycoside hydrolase enzymes possess, and contains putative catalytic amino acid residues (Asp-166, Glu-167, and Glu-222) in the active site^131^. The hydrolase domains of Sph3 and PelA (Sph3_h_ and PelA_h_, respectively) share structural and functional similarities given their ability to degrade GAG and disrupt preformed *Aspergillus fumigatus* biofilms in vitro^132^. A mechanistic study revealed that both Sph3_h_ and PelA_h_ function as retaining endo-α-(1,4)-*N-*acetylgalactosaminidases producing a minimal substrate size of seven residues^132^. *Ega3* is another gene in the GAG biosynthesis cluster encoding a putative α-(1,4)-galactosaminidase belonging to glycoside hydrolase family 114 (GH114) which also has the (β/α)_8_ fold structure; its activity depends on the conserved acidic residues, Asp-189 and Glu-247^133^. Recombinant Ega3 is an *endo*-acting α-(1,4)-galactosaminidase that disrupts GAG-dependent *A. fumigatus* and Pel polysaccharide-dependent *P. aeruginosa* preformed biofilms in vitro at nanomolar concentrations^133^.

#### β-(1,3) glucan glycoside hydrolase

β-(1,3) glucanases, which belong to the pathogenesis-related-2 family (PR-2), are abundant in nature and have been characterized from a wide range of species^134^. They successively cleave at the nonreducing end of β- (1,3) glucan producing oligosaccharides and glucose^134^. β-glucanase derived from *Arthrobacter luteus* is able to degrade poly-β-(1,3)-glucose in *Candida albicans* preformed biofilms in vitro but has no effect on planktonic growth or adhesion^101^.

#### Fructan glycoside hydrolase—levanase

Levanase, SacC, is an *exo*-fructosidase belonging to Glycoside Hydrolase Family 32 (GH32) and hydrolyzes the terminal β-(2,1)- d-fructofuranose residues of fructans from the non-reducing end^135^. Levanase SacC is able to hydrolyze both levan and inulin to produce fructose, and is also able to hydrolyze sucrose and raffinose^135^. Levanase cannot be detected in the wild-type *Bacillus subtilis*, but levanase SacC could be found in the culture medium of laboratory grown *B. subtilis* in the form of SacL mutated extracellular enzymes^136^. A recent study shows that extracellular levanase SacC from *B. subtilis* disrupts preformed *P. aeruginosa* biofilms in vitro, increasing the efficiency of conventional the antibiotics, ciprofloxacin and amikacin^137^.

#### Fructan glycoside hydrolase—inulinase

Inulinase is an enzyme that catalyzes the hydrolysis of β-(2,1)-d-fructosidic linkages in inulin and is part of a group of naturally occurring polysaccharides^138^. Inulinase can be subcategorized into exo-inulinase (EC 3.2.1.80) and endo-inulinase (EC3.2.1.7) based on hydrolysis patterns. Exo-inulinase hydrolyzes the terminal fructose residue of inulin from the non-reducing end, whereas endo-inulinase initiates hydrolysis at random positions within inulin to give fructooligosaccharides^139^. Inulinase is capable of degrading in vitro pre-formed biofilms on reverse osmosis RO membranes by composed by multiple bacterial species. The mechanism of its destructive process is degrading the β-(2, 6)-glucan fructosidic bonds of inulin^140^ (Table 1).

**Table 1:** Summary of glycoside hydrolase enzymes as biofilms dispersing agents.

Glycoside Hydrolase	Target	Summary
Dispersin B (DspB)	dPNAG	1. Glycoside hydrolase family 20 (GH20). 2. Hydrolyzes dPNAG via a substrate-assisted mechanism and has both endo- and exo-glycoside hydrolase activity. 3. Disperses preformed mature biofilms of various Gram-positive and Gram-negative bacterial species. DspB_E248Q_ is more active than the wildtype. 4. Commercialized as wound gel by Kane Biotech Inc, Dispersin B®.
PgaB	dPNAG	1. Contains a C-terminal CAZy GH153 family of glycosyl hydrolase that catalyzes the endoglycosidic cleavage of dPNAG. 2. Disrupts PNAG-dependent preformed mature biofilms by *B. pertussis, S. carnosus, S. epidermidis*, and *E. coli*.
Alginate Lyase	Alginate	1. Alginate lyase derived from *F. multivorum* with polyM/G activity is effective in dispersing preformed mature biofilms and has a synergistic effect with antibiotics. 2. Marine alginate lyase enzyme (AlyP1400) degrades preformed *P. aeruginosa* biofilms and enhances the bactericidal activity of tobramycin by modulating expression of efflux antibiotic resistance-related genes.
PslG_h_	Psl	1. Glycoside hydrolase family 39 (GH39). 2. The glycol-hydrolase domain of PslG, is a periplasmic glycoside hydrolase encoded by the Psl biosynthetic operon. 3. Inhibits biofilm formation of clinical and environmental isolates of *P. aeruginosa* over a 24-h period and is capable of disrupting newly formed biofilms but is less potent to against mature biofilms. 4. PslG_h_ is noncytotoxic and supports host immune responses.
PelA_h_	Pel GAG	1. PelA_47–303_ is constructed from the glycoside hydrolase domain of PelA which is a periplasmic enzyme encoded by the Pel biosynthetic operons. 2. PelA_h_ inhibits biofilms formed by clinical and environmental isolates of *P. aeruginosa* over a 24-h period. It is capable of disrupting newly formed biofilms but is less potent against disperse mature biofilms. 3. Boosts the efficacy of colistin and increases neutrophil killing by ~50%. 4. Shares structural and functional similarities with Sph3_h_, allowing degradation of GAG and disruption of preformed *A. fumigatus* biofilms in vitro.
Amylase	Amylose	1. Glycoside hydrolase family 13 (GH13). 2. Cleaves α-(1,4)-d-glucosidic linkages at random sites of amylose in biofilms. 3. Has a strong inhibiting effect on forming biofilms and degradation of pre-existing biofilms. 4. β-amylase, an exo-acting GH14 carbohydrolase, is less potent to disrupt biofilms. It hydrolyzes the α***-***(1,4)-glucosidic linkages of amylose only from the nonreducing end.
Cellulase	Cellulose	1. Cellulase from various sources, such as *P. funiculosum* and *T. reseei*, inhibits biofilm formation of *P. aeruginosa* in a pH dependent manner but is less effective for mature biofilms. 2. Cellulase combined with levofloxacin can powerfully disperse mature biofilms formed by *B. CalmetteGuerin*.
Sph3_h_	GAG	1. Glycoside hydrolase family 135 (GH135). 2. Glycol-hydrolase domain (Sph3_h_) of Sph3 is encoded by the GAG biosynthesis gene. 3. A retaining endo-α-(1,4)-*N-*acetylgalactosaminidase that produces a minimal substrate size of seven residues. 4. Degrades GAG and disrupts preformed *A. fumigatus* biofilms in vitro.
Ega3	GAG	1. Glycoside hydrolase family 114 (GH114). 2. Encoded by the GAG biosynthesis cluster, it is an endo-acting α-(1,4)-galactosaminidase. 3. Disrupts GAG-dependent *A. fumigatus* and Pel-dependent *P. aeruginosa* preformed biofilms in vitro at nanomolar concentrations.
(1,3)-β-Glucanase	(1,3)-β-Glucan	1. Exo-acting glycohydrolase, pathogenesis-related-2 family (PR-2), cleaving at the nonreducing end of β-(1,3) glucan oligosaccharides and glucose. 2. Derived from *A. luteus* and degrades poly-(1,3)-glucose in *C. albicans* preformed biofilms in vitro.
Levanase SacC	Levan Inulin Sucrose Raffinose	1. Exo-fructosidase in glycoside hydrolase family 32 (GH32). Hydrolyzes terminal β-(2, 1)-d-fructofuranose residues of fructans from the non-reducing end. 2. Can be procured in the culture medium of *B. subtilis* SacL mutants as extracellular enzymes.
Inulinase	Inulin	1. Hydrolyzes β-(2, 1)-d-fructosidic linkages in inulin. Subcategorized into exo- and endo-inulinase based on hydrolysis patterns. 2. Degrades in vitro pre-formed biofilms on reverse osmosis membrane comprised by multiple bacterial species.

### Proteases

Exoproteins, another major component of the EPS, is account for a considerable portion of the biomass of most biofilms. Exoproteins are crucial to bacterial cell aggregation, surface adhesion, and structural integrity of biofilm matrices^141,142^. Enzymatic degradation of EPS exoproteins is one of the most effective ways to eradicate biofilms. To date, a number of proteases capable of biofilm dispersal have been discovered and investigated.

#### Proteinase K

Proteinase K is a broad-spectrum serine protease with a wide pH tolerance (pH 4 - 12) and thermostability (37 - 60°C)^143^. It specifically cleaves peptide bonds in proximity to carboxylic groups of aliphatic and aromatic amino acids^144^. Proteinase K is capable of inhibiting *S. aureus* biofilm formation by hampering early adhesion, but also disperses 24-h- and 48-h-old biofilms^144^. Recent studies show that co-treatment of proteinase K with antibiotics has a synergistic effect that thoroughly degrades preformed biofilms produced by a range of bacteria, including *S. aureus*, *E. coli, Staphylococcus lugdunensis, Staphylococcus heamolyticus*, *Listeria monocytogenes*, *Gardnerella vaginalis*, and *Bdellovibrio bacteriovorus*^144–149^.

#### Trypsin

Trypsin, a pancreatic serine protease that specifically acts on the carboxyl side of lysine and arginine, has been applied to disperse biofilms formed on teeth and wounds^150–152^. Bovine trypsin can degrade mature biofilms of various Gram-positive and Gram-negative bacterial species^153^. Trypsin alone is able to reduce the biomass of the preformed 24-h-old biofilms of both *P. aeruginosa* and *Enterococcus faecalis* but cannot completely remove biofilms regardless of the treatment time and enzyme concentrations^154^. However, trypsin combined with pepsin and Carvacrol is able to fully disperse mature biofilms of *P. aeruginosa* and *E. faecalis* on various abiotic surfaces^154^.

#### Pepsin

Pepsin is a promiscuous endopeptidase with a catalytic aspartate in its active site to favorably cleave Phe and Leu residues; however, His, Lys, Arg, and Pro residues prohibit cleavage^155^. Pepsin reduces the biomass of the preformed 24-h-old biofilms of both *P. aeruginosa* and *E. faecalis*, but cannot completely remove biofilms from polystyrene surfaces regardless of the treatment time and enzyme concentrations used, like trypsin^154^. Co-administered with trypsin and carvacrol, it can effectively irradicate preformed *P. aeruginosa* and *E. faecalis* biofilms^154,156^.

#### Aureolysin

Aureolysin (Aur), an *S. aureus* expressed extracellular metalloprotease, down-regulates the formation of biofilms and allows for the mobility of bacteria by cleavage of surface binding proteins, such as clumping factor B which causes loss of fibrinogen binding in *S. aureus*^157^. Aur is a major contributor to bacterial pathogenicity via cleaving components of the innate host immune system and regulating bacterial toxins and cell wall proteins^158,159^. Aur is associated with the processing of other biofilm proteases, such as V8, SspB, and ScpA which together are known as the *Staphylococcal* proteolytic cascade. These proteases are secreted into the environment with the pro-peptide inhibiting their activation. Aur undergoes autocatalysis and becomes active by the degrading the pro-peptide, then mature aur cleaves the pro-peptide from V8 to generate active V8 protease. Finally, V8 will cleave the SspB pro-peptide to complete cascade^48^. Purified aur suppresses biofilm formation and disperses established biofilms of various *S. aureus* strains^160^.

#### V8 serine protease

The V8 serine protease, also known as SspA protease, is the major extracellular protease secreted by *S. aureus*. It is secreted as a proenzyme before being proteolytically cleaved by aur to become the mature V8 enzyme^48^. Research shows that V8 serine protease added at the beginning of cell culture prevents the *S. epidermidis* biofilm formation by degrading Bap protein, a surface-anchored protein^161^. Esp protease, produced by *S. epidermidis*, is structurally highly similar to that of V8. Purified Esp protease prevents biofilm formation, promotes disassembly of pre-established biofilms by cleaving autolysin (Atl)-derived murein hydrolases, and prevents *staphylococcal* release of extracellular DNA^49^.

#### Staphopain A

Staphopain A (ScpA), encoded by the *scpAB* operon, is also a participant of the *staphylococcal* proteolytic cascade. It is an extracellular cysteine protease generated by *S. aureus* and demonstrates a very broad range of substrate specificity^162^. Purified ScpA inhibits *S. aureus* formation and disperses established biofilms. The antibiofilm properties of ScpA are conserved across *S. aureus* strain lineages. Additionally, inhibition of ScpA restores the biofilm forming capacity of the biofilm-negative *S. aureus* mutant, the sigma factor B (ΔsigB) mutantt^163^. Purified ScpA enzyme inhibited *S. aureus* formation as well as to disperse the established biofilms, and the antibiofilm properties of ScpA were conserved across S. aureus strain lineages^163^.

#### Staphopain B

Staphopain B (SspB) is encoded by the *sspABC* operon and is a cysteine protease secreted by *S. aureus*. SspA cleaves proSspB to activate SspB in the last step of the *staphylococcal* proteolytic cascade^48^. Silencing expression of SspB can enable the biofilm-deficient *S. aureus* mutant (ΔsigB) to gain biofilm-forming abilities^163^. However, bacteria-derived proteinase could facilitate the bacterial colony survival by degrading antimicrobial peptides (AMPs) generated by the host immune system, which leads to chronic infections^164^. Fragments of the AMP, cathelicidin LL-37, have been discovered as part of the innate immune response in skin diseases such as atopic dermatitis and acne rosacea^164^. *S. aureus* derived proteinases aureolysin, V8, and SspB have been observed in *staphylococcal* isolates from atopic dermatitis patients and contributed to bacterial virulence through degradation of the AMP cathelicidin LL-37, resulting in loss of inhibitory activity on biofilm formation and ultimately leading to bacterial persistence in atopic dermatitis^165^.

#### SplABCDEF

Spl proteases consists of six serine proteases, SplABCDEF, encoded by the *νSaβ* gene in *S. aureus*^166^. Spl proteases modulate *S. aureus* physiology and virulence, and can induce disseminated lung damage during pneumonia likely by degrading surface-associated proteins in *staphylococcal* and human proteins^166^. In *S. aureus*, deletion of the genes encoding the extracellular proteases, aureolysin and Spl, encourages biofilm formation in planktonic cells^167^. These findings indicate that Spl proteases have the ability to disperse *S. aureus* biofilms, but more research is needed to elucidate the dispersal mechanism.

#### Surface protein-releasing enzyme (SPRE)

Endogenous surface protein-releasing enzyme (SPRE), produced by *S. mutans* NG8, can disperse the preformed monolayer biofilm of *S. mutans* and detach cells from colonized surfaces^168^. SPRE cleaves the bacterial surface anchoring protein, adhesin P1, by dissociating the bonds between the C-terminus of adhesin P1 and other cell surface components^169^. SPRE degrades preformed biofilms in a pH-dependent manner with the optimal pH range from 5 to 6, and can also detach biofilms of non-dividing cells, indicating that cells detached from biofilms were not daughter cells^168^.

#### *Streptococcal* cysteine protease (SpeB)

*Streptococcal* cysteine protease (SpeB) is secreted by *Streptococcus pyogenes*, an exclusively Gram-positive human pathogen that causes a wide spectrum of diseases such as pharyngitis, impetigo, toxic shock, and necrotizing fasciitis^170^. SpeB is a promiscuous enzyme displaying a broad range enzymatic activities including degradation of biofilm, cytokines, chemokines, complement components, immunoglobulins, and serum protease inhibitors. It is also capable of degrading and releasing other *streptococcal* proteins from the bacterial surface^170^. The constitutive production of SpeB by an *S. pyogenes* mutant strain is responsible for a significant reduction of biofilm formation. Beyond this, addition of purified SpeB to actively growing wild-type cultures significantly inhibits biofilm formation^171^. SpeB disperses biofilms and facilitates bacterial colonization and occupation of new areas, resulting in infections caused by *S. pyogenes* to vary from mild to severe^172^. However, a recent study shows that SpeB exhibits potent activity towards biofilm disruption at multiple stages of *S. aureus* biofilm formation by cleaving SdrC adhesin, which renders the bacteria more susceptible to antimicrobial agents and host immune components^173^.

#### Peptidase M16

Peptidase M16 is a *Microbacterium* sp. SKS10 secreted metalloprotease that exhibits optimal activity at 60°C, pH 12^174^. Peptidase M16 shows low cytotoxicity and excellent stability in the presence of various salts and organic solvents. Besides this, peptidase M16 can disperse mature *S. aureus* biofilms at concentrations lower than trypsin and α-amylase, and can be co-treated with kanamycin to enhance antimicrobial efficacy^174^ (Table 2).

**Table 2:** Summary of proteases as biofilm dispersing agents.

Protease	Summary
Proteinase K	1. Broad-spectrum serine protease with remarkable pH tolerance and thermostability. 2. Inhibits new biofilm formation and disperses preformed biofilms of various bacterial strains.
Trypsin	1. Pancreatic serine protease that specifically acts on the carboxyl side of lysine and arginine. 2. Degrades mature biofilms of various Gram-positive and Gram-negative bacterial species.
Pepsin	1. Promiscuous endopeptidase with a catalytic aspartate in its active site to favorably cleave Phe and Leu residues. 2. Reduces the biomass of preformed biofilms of both *P. aeruginosa* and *E. faecalis*.
Aureolysin (Aur)	1. *S. aureus* expressed extracellular metalloprotease. 2. Purified aureolysin can suppress biofilm formation and disperse established biofilms of various *S. aureus* strains.
V8 serine protease (V8) (SspA)	1. The main extracellular protease secreted by *S. aureus*. 2. Prevents formation of *S. epidermidis* biofilms by degrading Bap, a surface-anchored protein.
Staphopain A (ScpA)	1. Extracellular promiscuous cysteine protease generated by *S. aureus*. 2. Purified ScpA inhibits *S. aureus* biofilm formation and disperses established biofilms.
Staphopain B (SspB)	1. Secreted cysteine protease by *S. aureus*. 2. Inhibition enables the biofilm-deficient *S. aureus* mutant (ΔsigB) to restore biofilm forming abilities. 3. Degrades the AMP, cathelicidin LL-37, resulting in loss of inhibitory activity on biofilm formation.
Spl proteases	1. Consists of six serine proteases, SplABCDEF, modulating *S. aureus* physiology and virulence. 2. Degrade surface-associated proteins in *S. aureus*, and deletion of the genes encoding the extracellular Spl proteases encourages biofilm formation.
Surface protein releasing enzyme (SPRE)	1. Produced by *Streptococcus mutans* NG8, it can disperse the preformed monolayer biofilm of *Streptococcus mutans*. 2. Cleaves the bacterial surface anchoring protein, adhesin P1.
*Streptococcal* cysteine protease (SpeB)	1. Secreted by *S. pyogenes*, a Gram-positive human pathogen causing a wide spectrum of diseases. 2. Disperse biofilms and facilitates bacterial occupation of new areas, leading to serious infections. 3. Exhibits potent biofilm disruptive activity at multiple stages of *S. aureus* biofilm formation by cleaving SdrC adhesin.
Peptidase M16	1. Metalloprotease secreted by *Microbacterium* sp. SKS10. 2. Disperses mature *S. aureus* biofilms at low concentrations and can be co-treated with kanamycin to enhance antimicrobial efficacy.

### Deoxyribonucleases

Extracellular DNA (eDNA) is a ubiquitous and vital structural component of the EPS with functions including microbial adhesion, cell signaling, gene transfer, and biofilm matrix stabilization^175–177^. Despite the importance of eDNA in bacterial biofilms, it had not attracted much attention until 2002 when Whitchurch et al. exogenously added DNase I to disperse biofilms and boost bactericidal efficiency when combined with antibiotics^46^. Since then, substantial work has been done to employ various DNases targeting eDNA to eradicate biofilm infections.

#### DNase I

DNase I is a widely used pancreatic endonuclease that specifically digests DNA. It is secreted in the extracellular environment to degrade both single-stranded and double-stranded DNA into oligonucleotides with 5′ monophosphate and 3′ hydroxyl DNA ends^178,179^. DNase I can disrupt the formation of both mono- and polymicrobial biofilms^179^. Biofilms formed in the presence of DNase I display reduced biofilm biomass, total bacterial biomass, decreased viability of bacteria, and decreased tolerance to antibiotics^180^. However, DNase I is more effective towards the destruction of rapidly growing biofilms. Newly established biofilms (up to 60-h old) were also dissolved by DNase I treatment, whereas more mature biofilms (over 84-h old) exhibited strong resistance to DNase I degradation. This is likely due to mature biofilms being strengthened by other substances such as exopolysaccharides and exoproteins, additionally, mature biofilms may have produced sufficient proteolytic exoenzymes to locally inactivate DNase I^46^. Besides this, recombinant human DNase I (rhDNase) has been clinically applied in cystic fibrosis patients to reduce the viscosity of purulent sputum^181^. rhDNase exhibits strong antibiofilm activity and reduces the antibiotic resistance of *S. aureus* and *S. epidermidis*^182^. Recent studies show that DNase I also presents a wide compatibility with various antimicrobial agents such as ceftazidime, proteinase K, and silver sulfadiazine^183–185^. DNase I-like protein 2 (DNase1L2), found in the human stratum corneum of the epidermis, is able to suppress *P. aeruginosa* and *S. aureus* biofilm formation, indicating that DNase1L2 is an innate antimicrobial defense of the epidermis^186^.

#### Nucleases Xds and Dns

Extracellular nucleases, Xds and Dns, are produced by *Vibrio cholerae* and act as virulence factors in an infant mouse cholera model^187^. Xds, a Mg^2+^ dependent nuclease, belongs to the protein family PF03372 and exerts both endo- and exonuclease activity^188^. Dns, also known as VcEndA, belongs to the endonuclease I superfamily and does not have a specific nucleic acid cleavage site^189^. These two extracellular nucleases can degrade both circular and linearized DNA within biofilms, and deletion of the genes encoding these nucleases results in increased biofilm formation^47^. It would be worth directly determining their biofilm dispersing activity since Xds and Dns are secreted enzymes with good stability.

#### Streptodornase

Streptodornase, also known as Varidase, is a commercial mixture of four DNase enzymes produced by *P. aeruginosa*, which reduces the viscosity of biofilm matrices by digesting the eDNA of biofilms^190^. An in vitro study found streptodornase is more active against the pre-formed biofilms of *P. aeruginosa* than DNase I, and has been successfully applied in *P. aeruginosa* focal infections, such as urinary tract infection^191^.

#### NucB

NucB, a biofilm-dispersing nuclease from the marine *Bacillus licheniformis* strain, also disperses newly formed biofilms by degradation of eDNA. It is a non-specific endonuclease belonging to the ββα metal-dependent nuclease subfamily^192^, and can degrade preformed biofilms of coagulase-negative *staphylococci*, *S. aureus*, and α-hemolytic *streptococci* isolated from chronic rhinosinusitis infections, offering a promising therapeutic approach for chronic rhinosinusitis patients^193^ (Table 3).

**Table 3:** Summary of deoxyribonucleases as biofilms dispersing agents.

Deoxyribonuclease	Summary
DNase I	1. Widely used pancreatic endonuclease specific to the digestion of both single-stranded and double-stranded DNA. 2. More effective at preventing biofilm formation, and disrupts newly formed biofilms better than mature biofilms. 3. rhDNase has been clinically applied in cystic fibrosis patients to combat bacterial infection.
Nuclease Xds	1. Secreted Mg^2+^ dependent nuclease produced by *V. cholerae* with both endo and exo activity. 2. Belongs to protein family PF03372 and can degrade both circular and linearized DNA within biofilms. 3. Deletion of its encoding gene increases biofilm formation.
Nuclease Dns (VcEndA)	1. Member of endonuclease I superfamily produced by *V. cholerae*. 2. Degrades both circular and linearized DNA within biofilms. 3. Deletion of its encoding gene increases biofilm formation.
Streptodornase (Varidase)	1. Mixture of four DNase enzymes produced by *P. aeruginosa*. 2. Actively disrupts in vitro preformed biofilms of *P. aeruginosa* and is used against *P. aeruginosa* focal infections.
NucB	1. ββα metal-dependent nuclease derived from marine *B. licheniformis* disperses preformed biofilms by degrading eDNA. 2. Degrades the preformed biofilms of bacterial strains isolated from chronic rhinosinusitis infections.

### Current barriers and future directions of biofilm-dispersing enzymes

Utilization of enzymes to disperse biofilms has been a popular research topic for decades, and a number of dispersing enzymes have proven effective for inhibiting biofilm formation in diverse in vivo animal infection models including wound, nasal cavity, lung, and urinary tract^108^. Further, the rhDNase I, Dornase alfa, has been applied via inhalation in cystic fibrosis patients to reduce the viscosity of purulent sputum by preventing the establishment of chronic *P. aeruginosa* infection through inhibiting biofilm formation^181^. DispersinB® wound gel developed by Kane Biotech Inc. showed positive results in biocompatibility and in vivo preclinical studies and has been undergoing human clinical trials since 2022^114^. However, in practice most enzymatic biofilm eradication assays are carried out by in vitro testing against monomicrobial biofilms instead of multispecies-based biofilms that occur in nature. Thus, advancing the research of biofilm-dispersing enzymes is bottlenecked by the lack of the reliable biofilm models that mimic the true complexity of microbial colonization in humans and the world we live in.

Scientists must ascertain the compatibility of these enzymes and antibiotics with prudence. In order to enhance the potency of biofilm eradication, biofilm dispersing enzymes are always co-administered with antimicrobial agents, thereby providing an entryway to allow the antibiotics reach to the bacteria^194^. However, antibiotics can impact biofilm dispersing enzymes, for example, *S. aureus* micrococcal nuclease activity is modulated by sub-minimum inhibitory concentrations of antibiotics such as erythromycin and doxycycline^195^. Besides this, antibiotics bearing varied pKa values may also influence the activity of dispersing enzymes, thus robust enzymes with greater ranges of pH tolerance are more likely to be regarded as potential candidates for co-administration^196^. In the early stages of development, it is necessary to systematically characterize dispersing enzymes using artificial substrates that may need organic solvents in the testing buffer^197^. In preparations for manufacture and storage, enzymes with better thermostability will be favored for therapeutic development^196^. Therefore, it is necessary to consider enzymatic stability; including by not limited to thermostability, pH tolerance, and durability in organic solvents; when developing such dispersal agents for clinical application. Biofilm-dispersing enzymes, such as peptidase M16 and proteinase K, demonstrate remarkable stability in organic solvents, a wide pH tolerance, and great thermostability (37-60°C)^143,174^, but not all enzymes can maintain activity under harsh conditions. For example, DspB will denature when over 5% DMSO is present in solution^106^, and SPRE disperses biofilm in a pH-dependent manner, ranging between pH 5 – 6^168^.

Toxicity is another issue to be addressed in the development of enzymatic biofilm dispersal approaches, as these exogeneous agents could elicit strong immune responses or exhibit cellular toxicity. In a study on glycosidases to degrade biofilm, a total of 12 glycol-hydrolases including alginate lyase, amylase, amyloglucosidase, xylanase, cellulase, and pectinase were found to be cytotoxic towards human epithelial fibroblasts and human normal colonic cells^108^. Furthermore, xylanase displayed harmful effects in wound tissue at the wound site and even negatively impacted the spleen^108^.

Additionally, dispersing enzymes cannot achieve bactericidal activity and even facilitate bacterial colonization of new areas, leading to new or ongoing infections^172^. Research even shows that aureolysin and staphopain are able to degrade AMPs produced by the host immune system, resulting chronic atopic dermatitis^172^. Thus, biofilm dispersal enzymes are always combined with an antibiotic or other therapies to combat bacterial infections and may come into clinical applications in the near future, but careful consideration must be placed into the selection of agents for co-administration. Scientists have utilized enzyme engineering coupled with high-throughput screening to discover new enzymes as biofilm-dispersing agents^106,107,140,198^.

In summary, the capability of traditional antibiotics has been greatly compromised in recent years by increasing antibiotic tolerance of biofilm-embedded microbial pathogens. Clinically, biofilm-associated infections account for around 80% of human bacterial infections. Thus, effective biofilm dispersal strategies have been extensively sought after, and enzymatic dispersal stands out from other biofilm degrading methods due to its efficiency and specificity without causing selective pressure on bacteria. Biofilm-dispersing enzymes can effectively break down the EPS, leading to a collapse of biofilm matrices and making microbial cells accessible to antibiotic treatments or host immune responses. In this review, we have summarized the three major families of biofilm dispersal enzymes; glycosidases, proteases, and deoxyribonucleases; which target biofilm exopolysaccharides, extracellular proteins, and eDNA, respectively. Although numerous enzymes with biofilm-dispersing abilities have been discovered and demonstrate promising results in vitro, only a few in vivo studies have been performed, with clinical trials conducted for even less enzymes. Issues like toxicity, compatibility, and stability of biofilm-degrading enzymes have not yet been fully addressed. Further efforts are needed to develop robust, safe, and potent biofilm-dispersing enzymes for clinical applications.
